# From Tradition to Innovation: The Role of Sea Fennel in Shaping Kimchi’s Microbial, Chemical, and Sensory Profiles

**DOI:** 10.3390/molecules30132731

**Published:** 2025-06-25

**Authors:** Maryem Kraouia, Maoloni Antonietta, Federica Cardinali, Vesna Milanović, Cristiana Garofalo, Andrea Osimani, Antonio Raffo, Valentina Melini, Nicoletta Nardo, Irene Baiamonte, Lucia Aquilanti, Giorgia Rampanti

**Affiliations:** 1 Department of Agricultural, Food, and Environmental Sciences (D3A), Università Politecnica delle Marche (UNIVPM), Via Brecce Bianche, 60131 Ancona, Italy; m.kraouia@pm.univpm.it (M.K.); a.maoloni@univpm.it (M.A.); f.cardinali@univpm.it (F.C.); v.milanovic@univpm.it (V.M.); c.garofalo@univpm.it (C.G.); a.osimani@univpm.it (A.O.); g.rampanti@univpm.it (G.R.); 2 CREA-Research Centre for Food and Nutrition, Via Ardeatina, 546, 00178 Rome, Italy; antonio.raffo@crea.gov.it (A.R.); valentina.melini@crea.gov.it (V.M.); nicoletta.nardo@crea.gov.it (N.N.); irene.baiamonte@crea.gov.it (I.B.)

**Keywords:** kimchi, sea fennel, lactic acid bacteria, starter culture, fermentation

## Abstract

Kimchi, a traditional fermented product made primarily with Chinese cabbage, develops its characteristic flavor through microbial activity and a variety of ingredients. This study explores the incorporation of sea fennel (*Crithmum maritimum* L.), a halophytic plant rich in bioactive compounds and known for its distinctive aroma, into kimchi. Two fermentation methods were compared: spontaneous fermentation and fermentation using a defined starter culture of four lactic acid bacteria strains. Fermentation was conducted at 4 °C for 26 days, with samples monitored for up to 150 days. Parameters analyzed included pH, titratable acidity, microbial counts, organic acid concentrations, volatile organic compounds (VOCs), and sensory attributes. In the early stages, notable differences in acidity, microbial populations, and VOCs were observed between the two methods, but these differences diminished over time. Sensory analysis indicated similar overall characteristics for both prototypes, although the sea fennel’s aroma and fibrous texture remained perceptible at day 150. VOCs analysis revealed that the fermentation time significantly affected the composition of key aroma compounds, contributing to the final sensory profile. Sea fennel played a key role in shaping the VOC profile and imparting a distinctive aromatic quality. Both fermentation methods led to similar enhancements in flavor and product quality. These findings support the use of sea fennel as an aromatic ingredient in fermented vegetables and highlight the importance of fermentation optimization.

## 1. Introduction

Fermentation is recognized as one of the oldest and most cost-effective methods for food production and preservation [1]. In ancient civilizations, fermented vegetables were used as substitutes for fresh produce during seasons of limited availability. A wide variety of fermented vegetables exist, each distinguished by the raw materials, recipes, and fermentation techniques employed [2]. Common examples include sauerkraut [3,4], paocai [5], zhacai [6], and kimchi [7].

Among the wide range of fermented vegetable products produced in Asia, kimchi stands out as the most iconic representative of Korean cuisine [8], with a history of consumption spanning over 2000 years [9]. Kimchi holds a central role in traditional Korean dishes and is considered one of the most flavor-rich fermented foods, not only in Korea but also in other East Asian countries such as Japan and China [8]. It is prepared using ingredients such as cabbage, radish, and cucumber, combined with seasonings like salt, red pepper powder, garlic, leek, and ginger [10]. Kimchi varieties are typically classified based on the ingredients used or the preparation methods applied [11]. The most popular type is Baechu Kimchi, made with Chinese cabbage [12]. With over 200 known varieties, kimchi represents a cultural convergence point across different regions of Korea and has inspired a strong scientific interest [13]. Previous studies have reported an average daily kimchi intake in Korea of 124.3 g, with individuals aged 30–49 consuming up to 154.5 g per day [11]. Kimchi is also nutritionally rich, containing vitamins (e.g., vitamin C, carotene, B-complex), minerals (e.g., calcium, iron, potassium), and dietary fiber [14]. Moreover, well-fermented kimchi offers a unique combination of sour, spicy, hot, sweet, and fresh flavors [15].

Kimchi is considered a “living food” due to the dynamic production of physiologically active compounds by diverse microorganisms naturally present in its ingredients, which initiate fermentation. This complex process is primarily driven by lactic acid bacteria and various enzymes [14,16]. Lactic acid bacteria are essential not only for fermentation but also for the preservation and maturation of the final product [17]. During fermentation, lactic acid bacteria produce organic acids that lower the pH from approximately 6.39 to 4.34 and increase acidity from 0.06% to 0.35%, contributing to product stability during storage [18,19]. In addition, lactic acid bacteria generate several metabolites including carbon dioxide, ethanol, vitamins, bacteriocins, prebiotic factors, and flavor compounds such as mannitol and amino acids, all of which enhance both health benefits and sensory characteristics [20,21,22].

The fermentation of kimchi is characterized by dynamic microbial succession, strongly influenced by environmental factors such as salt concentration. A concentration of 1.5–4% promotes the proliferation and dominance of lactic acid bacteria, despite their initially low levels in raw ingredients [10]. Temperature also plays a key role in microbial activity: strains from the genera *Leuconostoc*, *Pediococcus*, and *Lactococcus* dominate early stages but decline at storage temperatures of 20–30 °C [23]. Lactic acid bacteria genera such as *Leuconostoc*, *Lactobacillus*, and *Weissella* are identified as key dominant groups during kimchi fermentation [10]. Furthermore, kimchi serves as a rich source of probiotics, which adapt well to fermentation due to their metabolic versatility, transforming the product into a functional food with multiple health benefits [24]. Certain lactic acid bacteria strains, such as *Lactobacillus plantarum* EM—previously isolated from kimchi, have shown promising probiotic potential, including the ability to reduce serum cholesterol levels [25].

The quality and shelf life of kimchi are closely linked to the microorganisms involved in fermentation. In natural fermentation, the process is initiated by the native microbiota present in raw materials, generating a complex and diverse microbial environment. The dominance of specific microorganisms shifts throughout fermentation, depending on growth conditions and the accumulation of metabolic by-products [26]. Fermentation by non-starter lactic acid bacteria can lead to variability in taste and sensory characteristics. Therefore, industrial kimchi production in Korea often employs defined starter cultures to better control the fermentation process and standardize product quality [10]. Consequently, the physicochemical and sensory attributes of kimchi produced via natural or guided fermentation may vary depending on environmental factors, microbial diversity, and raw ingredients. Comparative studies between these fermentation methods can help elucidate the impact of starter cultures on kimchi quality and sensory outcomes.

Sea fennel (*Crithmum maritimum* L.) is a perennial, highly aromatic halophyte native to the Mediterranean coast. It is rich in bioactive compounds—including vitamin C, polyphenols, carotenoids, and fatty acids—and is known for its potential to prevent chronic degenerative diseases [27,28]. Sea fennel has gained attention as a promising plant-based ingredient for the development of innovative foods, food additives, and nutraceuticals [29]. Previous studies have identified it as an emerging crop with strong potential for food innovation, particularly in high-value products such as fermented preserves and spices [28,30,31]. Moreover, sea fennel has been recognized as an effective carrier for probiotics, facilitating their survival during gastrointestinal transit [32].

The aim of this study was to investigate the incorporation of sea fennel into non-Korean kimchi preparations. Two laboratory-scale prototypes—one produced via spontaneous fermentation and the other via fermentation with a defined lactic acid bacteria starter culture—were developed, using Chinese cabbage as the primary ingredient and sea fennel as a flavor-enhancing component. These prototypes were evaluated for their physicochemical characteristics, microbial dynamics, and key sensory and volatile profiles, both at the end of fermentation and after 150 days of storage.

## 2. Results

### 2.1. Measurement of pH

The pH values measured in starter-inoculated and naturally fermented kimchi are presented in Table 1.

Both prototypes followed a similar overall trend, with no significant differences observed during the early stage of fermentation. However, the starter-inoculated kimchi exhibited a more rapid decrease in pH compared to the naturally fermented control. Specifically, the pH dropped from 5.20 ± 0.07 to 4.90 ± 0.10 between days 2 and 7, while the control sample showed a more gradual decline, particularly during the middle phase of fermentation. By the end of the fermentation period, no significant differences were observed between the final pH values of the two prototypes, which reached 3.97 ± 0.01 for the control and 3.90 ± 0.02 for the starter-inoculated kimchi.

### 2.2. Determination of Titratable Acidity (TA)

The results of the TA measurements are shown in Table 2.

An inverse trend was observed compared to pH values. Although TA followed a similar pattern in both prototypes, the starter-inoculated kimchi exhibited a slightly higher final value at the end of fermentation, reaching 0.53 ± 0.05% lactic acid equivalent, compared to 0.34 ± 0.03% in the naturally fermented kimchi.

### 2.3. Organic Acids Quantification

The results of organic acids quantification for both kimchi samples are presented in Table 3.

The total lactic acid content increased significantly in both the starter-inoculated and naturally fermented kimchi throughout the fermentation process. In the control sample, lactic acid levels rose from 0.02 ± 0.00 to 0.56 ± 0.04 g 100 g^−1^, while in the starter-inoculated kimchi, they increased from 0.03 ± 0.00 to 0.52 ± 0.01 g 100 g^−1^. After 150 days of storage, a further increase in lactic acid content was observed in the starter-inoculated kimchi, reaching 0.69 ± 0.15 g 100 g^−1^. In both samples, the L-lactic acid isomer was more abundant than the D-isomer.

In contrast, the acetic acid concentration at the end of fermentation was significantly higher in the starter-inoculated kimchi (0.10 ± 0.02 g 100 g^−1^) compared to the control (0.02 ± 0.01 g 100 g^−1^). However, after 150 days, the acetic acid concentration in the naturally fermented kimchi significantly increased to 0.10 ± 0.02 g 100 g^−1^, while in the starter-inoculated sample, it showed a slight increase, reaching 0.13 ± 0.04 g 100 g^−1^.

In conclusion, both prototypes exhibited a consistently higher yield of lactic acid compared to acetic acid, both at the end of fermentation and after 150 days, confirming the predominance of lactic acid fermentation in both processes.

### 2.4. Microbial Enumeration

The results of viable cell counts in starter-inoculated and control kimchi samples are presented in Table 4.

Initially, the load of mesophilic lactobacilli was significantly lower in the control sample compared to the starter-inoculated kimchi. However, from day 12 onward, both prototypes exhibited a similar trend in the growth of mesophilic aerobic bacteria and mesophilic lactobacilli, maintaining comparable microbial loads by the end of fermentation, with values ranging between 8.2 ± 0.1 and 8.6 ± 0.1 Log CFU g^−1^.

As for mesophilic lactococci, their population was significantly lower in naturally fermented kimchi throughout the fermentation period compared to the starter-inoculated sample. At the end of fermentation, mesophilic lactococci reached 6.1 ± 0.4 Log CFU g^−1^ in the control and 8.3 ± 0.1 Log CFU g^−1^ in the starter-inoculated kimchi.

Yeast counts remained below the detection threshold (<1.0 Log CFU g^−1^) throughout the entire fermentation process in both prototypes.

Similarly, no statistically significant differences were observed in the population of *Pseudomonadaceae* between the starter-inoculated and control kimchi samples, except at t_12_. However, a marked decrease was observed in both cases by the end of fermentation, with final values of 2.7 ± 0.6 and 2.1 ± 0.4 Log CFU g^−1^ for the naturally fermented and starter-inoculated kimchi, respectively.

Regarding *Enterobacteriaceae*, no significant differences were detected between the two fermentation methods. In both prototypes, bacterial loads decreased below the detection limit (<1.0 Log CFU g^−1^) by the end of fermentation.

*Listeria monocytogenes* was not detected in 25 g of product. Moreover, coagulase-positive *Staphylococcus* spp. and sulfite-reducing bacteria were below the detection limit (<1 CFU g^−1^) at both the end of fermentation and after 150 days of storage.

### 2.5. Volatile Organic Compounds (VOCs) Analysis

The results of the semi-quantitative analysis of VOCs detected in the static headspace of the two kimchi prototypes made with sea fennel are reported in Table 5. A total of 38 compounds were identified, including (i) aldehydes (4): acetaldehyde, pentanal, geranial, neral; (ii) esters (3): ethyl acetate, 1-butanol-3-methyl acetate, 1-methoxy-2-propyl acetate; (iii) alcohols (6): ethanol, 1-penten-3-ol, 3-methyl-1-butanol, (Z)-3-hexen-1-ol, 1-pentanol, phenylethyl alcohol; (iv) carboxylic acids (3): acetic acid, hexanoic acid, octanoic acid; (v) terpenes (15): camphene, α-pinene, β-myrcene, sabinene, γ-terpinene, D-limonene, β-phellandrene, p-cymene, α-terpineol, terpinene-4-ol, borneol, 1,8-cineole, camphor, thymol methyl ether, dill apiole; (vi) sulfur compounds (2): dimethyl trisulfide, dimethyl disulfide; (vii) nitriles (2): 5-cyano-1-pentene, benzenepropanenitrile; (viii) phenols (1): carvacrol; (viii) heterocyclic compounds (1): unknown thiazole; and (ix) isothiocyanates (1): phenethyl isothiocyanate.

The VOC profile showed a generally similar trend in both the control and starter-inoculated kimchi, with only minor variations in specific compounds. Acetaldehyde, present at the beginning of fermentation (t_0_), was no longer detectable at the end of fermentation (t_26_) or after 150 days (t_150_). Similarly, sabinene showed high levels at t_0_ but decreased significantly to negligible levels in both samples.

During fermentation, concentrations of dimethyl disulfide, acetic acid, dill apiole, α-terpineol, borneol, benzenepropanenitrile, p-cymene, 4-ethyl-5-methylthiazole, terpinene-4-ol, and phenethyl isothiocyanate increased significantly from t_0_ to t_26_. Among these, *p*-cymene and terpinene-4-ol were the most abundant, with terpinene-4-ol reaching peak areas of 239.30 ± 41.02 and 249.58 ± 28.29 × 10^5^ in the control and starter-inoculated kimchi, respectively, at t_26_. Dill apiole was more abundant in the control sample, while acetic acid was higher in the starter-inoculated one.

At t_150_, further increases were observed in several VOCs, including ethanol, 1-pentanol, carboxylic acids (hexanoic and octanoic acids), 3-methyl-1-butanol, ethyl acetate, and terpinene-4-ol. Concentrations in the control sample reached 175.14, 275.84, and 451.32 × 10^5^ for ethanol, 3-methyl-1-butanol, and terpinene-4-ol, respectively, compared to 124.80, 200.60, and 333.30 × 10^5^ in the starter-inoculated kimchi.

Several compounds, including γ-terpinene, β-myrcene, camphor, thymol methyl ether, D-limonene, and 1-methoxy-2-propyl acetate, remained stable throughout fermentation. Similarly, phenolic compounds such as carvacrol showed minimal variation over the entire monitoring period.

**Figure 1:**
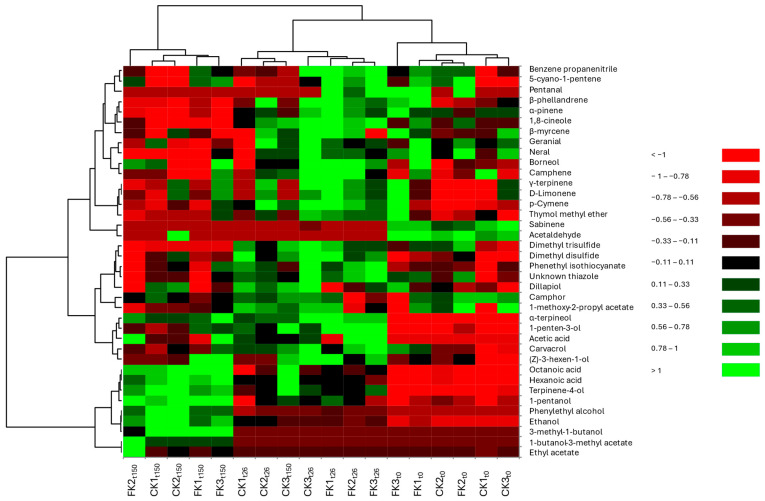
Plot of heatmap analysis performed on control and started kimchi made with sea fennel (38 VOCs in 18 Kimchi samples). Sample labels indicate treatment group (started Kimchi, FK, or control Kimchi, CK), replicate number (1, 2, or 3), and sampling time (t_0_, t_26_, or t_150_). The color gradient from red to green represents relative VOC abundance, with red indicating low levels and green indicating high levels.

A heatmap was generated to visualize the concentration of VOCs across replicates and fermentation times (Figure 1).

Low concentrations are represented in red, while high concentrations appear light green. The heatmap showed clustering of samples based on fermentation stage: t_0_ samples clustered together, as did most t_26_ and t_150_ samples—except for CK5 (t_150_), which displayed a distinct profile. At t_0_, acetaldehyde and sabinene were predominant, regardless of the use of starter cultures. By t_26_, significantly higher concentrations of terpenes (β-phellandrene, α-pinene, D-limonene, *p*-cymene) and acetic acid were detected in most replicates, indicating their accumulation over time. At t_150_, both prototypes exhibited enriched profiles of terpinene-4-ol; alcohols such as (Z)-3-hexen-1-ol, 1-pentanol, 3-methyl-1-butanol, and ethanol; and carboxylic acids including octanoic and hexanoic acid in samples FK1, FK2, FK3, CK1, and CK2. Additional relevant compounds at this stage included ethyl acetate and 3-methyl-1-butanol.

**Figure 2:**
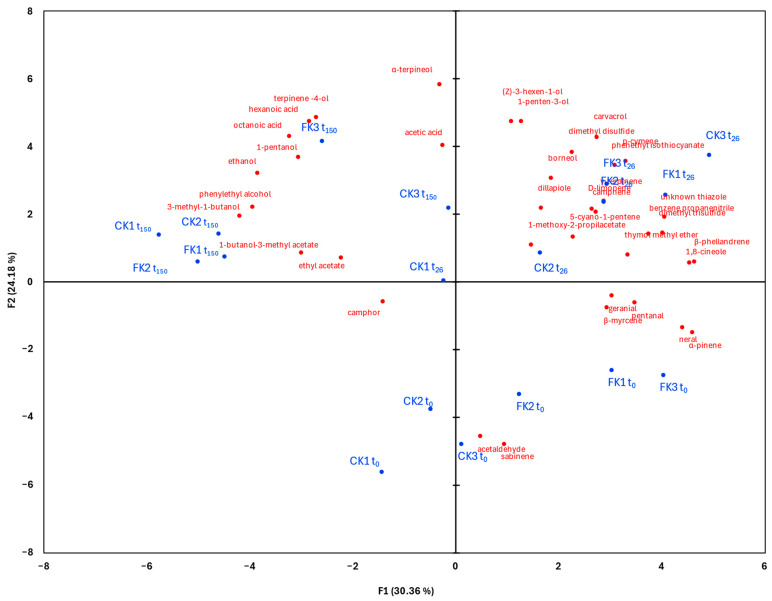
Principal component analysis biplot of the first two PCs obtained on VOCs dataset. Observations, in blue labels, refer to kimchi samples: labels indicate the type of kimchi (FK for starter-inoculated kimchi, CK for control kimchi), followed by replicate numbers (1, 2, 3) and sampling time (t_0_, t_26_, t_150_). Variables, in red labels, refer to individual VOCs.

To explore the relationships between VOCs, fermentation time, and treatment, a principal component analysis (PCA) was conducted. Figure 2 shows the biplot of the PCA score and loading plots. Principal components 1 and 2 (PC1 and PC2) explained 30% and 24% of the total variance, respectively, accounting for a cumulative 54.54% of the data variability.

PC1 effectively separated t_150_ samples and specific earlier-stage samples (e.g., CK1 t_26_, CK2 t_0_, and CK1 t_0_) with negative scores from most t_0_ and t_26_ samples with positive scores. PC2 differentiated early fermentation samples (t_0_, with negative scores) from those at t_26_ and t_150_. The clustering pattern revealed that the fermentation stage had a greater influence on the VOC profile than the use of starter culture.

The metabolite distribution across fermentation stages indicated that aldehydes and monoterpenes were dominant at t_0_, whereas phenols, alcohols, esters, and carboxylic acids were associated with t_26_ and t_150_ samples. Specifically, t_0_ samples (CK1, CK2, CK3, FK1, FK2, FK3) were associated with aldehydes and monoterpenes. By t_26_, samples (CK2, CK3, FK1, FK2, FK3) were linked to terpenes, alcohols, sulfur-containing compounds, and phenols. The sample CK2 t_26_ displayed a unique VOC profile compared to the other control replicates. At t_150_, the VOC profiles in both treatments were characterized by higher levels of carboxylic acids (acetic, octanoic, hexanoic) and alcohols (ethanol, 3-methyl-1-butanol, 1-pentanol), indicating continued metabolite evolution during storage.

### 2.6. Sensory Evaluation

**Figure 3:**
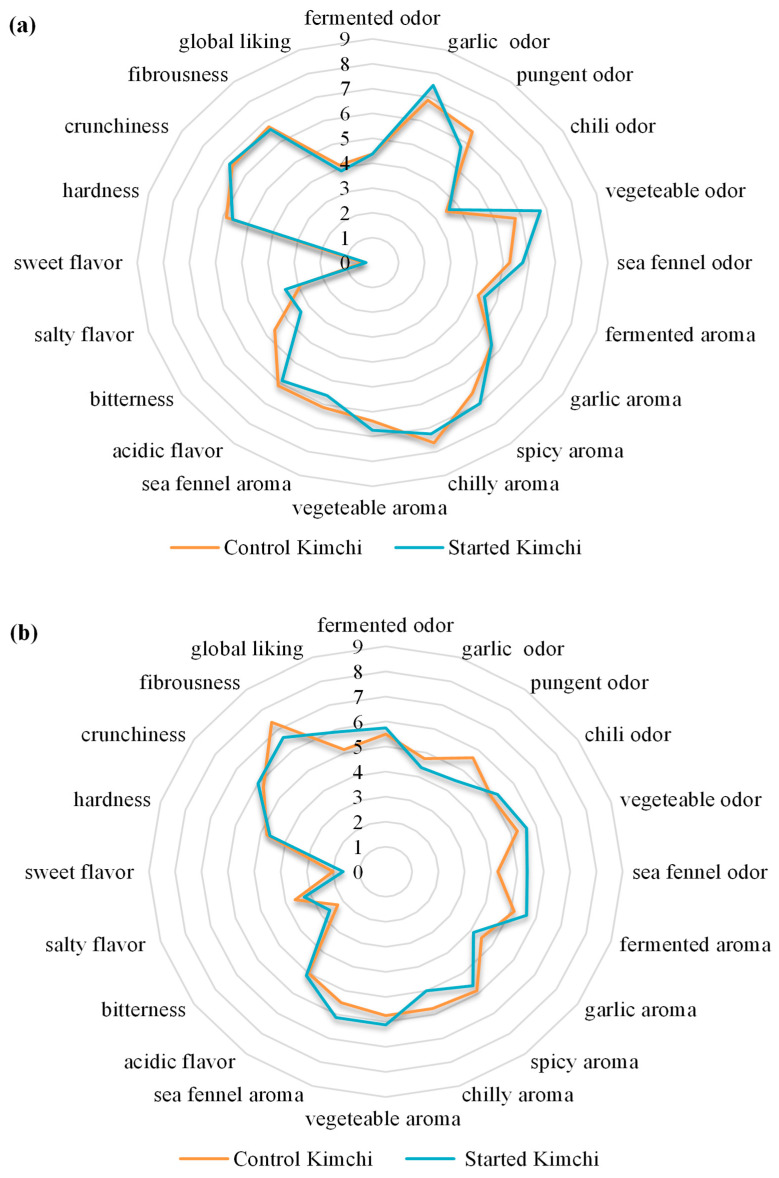
Sensory evaluation of started and control kimchi. (**a**) Sensory analyses performed after 26 days of fermentation; (**b**) Sensory analyses performed after 150 days since the start of fermentation. Each sample was evaluated by a trained panel, consisting of 8 non-smoker tasters aged between 25 and 48, for the presence and intensity of (i) six olfactory descriptors, being fermented, garlic, pungent, chilly, vegetable, and sea fennel; (ii) six aroma descriptors, being fermented, garlic, spicy, chilly, vegetable, and sea fennel; (iii) four flavor descriptors, being acidity, bitterness, salty, and sweet; (iv) three textural descriptors, being hardness, fibrousness, and crunchiness; and (v) global acceptance. Each descriptor was evaluated by attributing a score comprised between 1 and 9, with 1 expressing the lowest and 9 the highest intensity. Results are reported as mean values ± standard deviation.

The results of the sensory analysis are presented in Figure 3.

Overall, most sensory attributes were perceived similarly between the two prototypes, regardless of the use of the starter culture. No statistically significant differences were observed between the control and started kimchi at any sampling time (*p* > 0.05). Spiciness, chili aroma, and garlic odor were more strongly perceived in all samples at day 26 (t26) (Figure 3, panel a) compared to day 150 (t150) (Figure 3, panel b). No significant differences in crunchiness were observed between the two samples at either time point; however, fibrousness slightly increased in both after 150 days of storage.

The sea fennel flavor was perceived as slightly more intense in the starter-inoculated kimchi than in the control at t150. Both samples received relatively low scores for sweetness and saltiness at both sampling times. Regarding overall acceptability, the starter-inoculated kimchi was rated more favorably than the naturally fermented counterpart at t150, with the highest score being 5.88 ± 0.64.

## 3. Discussion

Kimchi fermentation is primarily driven by various lactic acid bacteria species naturally present in raw materials, whose activity is influenced by several factors, including ingredient composition, fermentation temperature, and salt concentration [33]. This study investigated the incorporation of sea fennel, a novel aromatic crop, as a flavoring ingredient in the production of non-Korean kimchi. Additionally, the fermentation dynamics of spontaneously fermented (control) kimchi were compared with those of kimchi inoculated with a selected multi-strain lactic acid bacteria starter culture. These lactic acid bacteria strains are known for their pro-technological relevance due to their ability to rapidly acidify the matrix, inhibit spoilage and pathogenic microorganisms, and enhance the safety and nutritional value of the final product.

Titratable acidity (TA) and pH were used as indicators of fermentation progress, and both parameters showed dynamic trends in line with previous findings [9]. Optimal fermentation conditions typically include a period of 20–30 days at 4–5 °C to achieve desirable pH and TA values [34]. In the present study, pH initially increased slightly between days 2 and 5—likely due to the release of water-soluble compounds such as minerals and nutrients from the vegetables—before undergoing a marked decline. The starter-inoculated kimchi exhibited a more rapid pH drop than the control, though both reached similar pH values by the end of fermentation (3.90 ± 0.02 for starter vs. 3.97 ± 0.01 for control), consistent with earlier observations [26,35].

Although a pH value of 3.9 is lower than the optimal range of 4.2–4.5 typically associated with high-quality kimchi [36,37], it still indicates a well-fermented product. This trend is consistent with findings by Jung et al. [38] and Lee et al. [39], who reported pH values of 4.3–4.7 after 24 days and approximately 4.2 after 5 weeks, respectively. The initial pH increase followed by a sharp drop and subsequent stabilization aligns with typical fermentation kinetics [39,40], driven by the production of organic acids—mainly lactic and acetic acid—through enzymatic and metabolic pathways employed by LAB, including the Embden–Meyerhof, phosphoketolase, tagatose-6-phosphate, and Leloir pathways [41,42].

Inversely to pH, titratable acidity increased over time, reflecting organic acid accumulation during fermentation [43]. By the end of the fermentation process, TA was significantly higher in starter-inoculated kimchi (0.53 ± 0.05% lactic acid equivalent) than in the control (0.34 ± 0.03%), confirming that lactic acid bacteria activity was more pronounced in the inoculated samples. The role of ingredients and environmental conditions in modulating this increase cannot be overlooked [8]. Generally, optimal taste is achieved when TA reaches 0.5–0.6% lactic acid equivalent [44], which aligns with the values observed in the starter-inoculated kimchi in this study.

Organic acids, especially lactic and acetic acid, are key metabolic products of lactic acid bacteria and serve as quality indicators for kimchi, contributing to its flavor and microbial safety [23]. These acids disrupt microbial cell walls and membranes, inhibiting spoilage and pathogenic bacteria [45]. The present study found a higher concentration of lactic acid compared to acetic acid in both prototypes, with the starter-inoculated kimchi showing slightly higher lactic acid levels. This finding is consistent with previous work [23,46], confirming the strong correlation between TA and lactic acid content. Lactic acid, as the dominant organic acid produced during fermentation, enhances kimchi’s characteristic sourness, improves microbial stability, and increases consumer acceptability [23,47].

Lactic acid bacteria population dynamics during fermentation have been widely reported to follow a pattern of initial growth followed by stabilization [35]. Viable counts revealed a progressive increase in LAB populations in both prototypes, accompanied by a decrease in spoilage-associated microorganisms, including yeasts, *Enterobacteriaceae*, and *Pseudomonadaceae*. However, the starter-inoculated kimchi showed initially higher counts of LAB and mesophilic aerobic bacteria than the naturally fermented sample, consistent with previous findings attributing this to starter cultures application [48].

In a study examining the influence of capsaicinoids on fermentation, Chung et al. [49] reported a rise in mesophilic aerobic bacteria after 14 days, reaching loads of 6.98–7.11 Log CFU g^−1^. Similarly, Cho et al. [50] suggested that increases in total aerobic bacteria during fermentation correlate with the accumulation of metabolic products such as lactate and acetate.

LAB are crucial in kimchi fermentation, as they produce organic acids such as lactic and acetic acid that inhibit spoilage and pathogenic bacteria [51]. Additionally, some lactic acid bacteria strains synthesize bacteriocins—antimicrobial peptides that suppress unwanted microbial growth. For example, *Lactococcus lactis* subsp. *lactis*, isolated from kimchi, produces nisin, which is effective against *Clostridium perfringens*, *Listeria monocytogenes*, and antibiotic-resistant strains such as MRSA and VRE [52]. Lactic acid bacteria also contribute to the synthesis of volatile compounds through amino acid metabolism, enhancing aroma development [53]. Accordingly, recent approaches suggest using multi-strain lactic acid bacteria starter cultures to accelerate fermentation and improve product quality [54].

In the current study, lactic acid bacteria counts differed significantly between treatments during the initial fermentation period. In the control sample, lactobacilli increased from 1.9 to 5.1 Log CFU g^−1^ within the first 5 days, reaching 8.2 Log CFU g^−1^ by day 12. In contrast, starter-inoculated kimchi began with a higher LAB load (~7.5 Log CFU g^−1^), which remained stable in the early stages. The initial microbial load observed in the control sample also aligns with previous findings [12,15,49]. Moreover, the lower lactic acid bacteria counts in the control sample may be partially attributed to antimicrobial compounds in sea fennel, such as essential oils and phenolics, which can inhibit certain lactic acid bacteria strains.

Despite initial differences, lactic acid bacteria populations converged by the end of fermentation, reaching ~8.4 Log CFU g^−1^ in both treatments. This trend confirms previous findings by Jung et al. [35], who reported similar stabilization of lactic acid bacteria populations, regardless of starter application. Comparable increases in LAB have also been described by Kim et al. [55] and Song et al. [56], who recorded counts of 7.86 Log CFU mL^−1^ and 5.46–5.75 Log CFU mL^−1^, respectively, during spontaneous fermentation.

Maoloni et al. [57] suggested that sea fennel could inhibit lactic acid bacteria growth due to its phenolic and bacteriocin content, as previously hypothesized by Zaika et al. [58]. Nonetheless, in the present study, LAB counts in starter-inoculated kimchi were not significantly suppressed, suggesting that the selected LAB strains were compatible with the sea fennel matrix.

The key compounds and volatile components produced during kimchi fermentation significantly contribute to the development of a product with an acceptable flavor [59]. In this study, the volatilome of kimchi made with sea fennel with or without starter culture was investigated. The results revealed a clear correlation between the volatile compounds and the progression of fermentation over time, regardless of the application of starter culture.

Volatile compounds are primarily formed through different chemical oxidation and enzymatic reactions during fermentation. Moreover, microorganisms contribute to the formation of VOCs using the substrates in kimchi, including constituents of cabbage, red pepper, garlic, and ginger [60]. Semi-quantitative analyses showed statistically significant changes in specific VOCs during fermentation, with time being the primary factor influencing the volatile profile.

Overall, the concentration of major volatile compounds showed increasing levels during the fermentation process. At the early stage (t_0_), terpenes were detected, with γ-terpinene being the most prominent. These findings align with those of Choi et al. [61], who reported that most of the terpenes were abundant at the early stage of fermentation. Acetaldehyde was detected at t_0_ in all replicates but was not detected in the later stages of fermentation. Previous studies have suggested that acetaldehyde decreases during fermentation due to the activity of microorganisms that convert acetaldehyde and other compounds, such as acetone, to ethanol as kimchi ripens [46]. Lee et al. [62] has also reported that aldehydes compounds tend to decrease or cannot be detected as fermentation progresses.

At t_26_, the volatile profile became more complex and diverse, exhibiting a wide range of compounds, including terpenes, nitriles, and sulfur-containing compounds. Terpenes such as p-cymene increased significantly from t_0_ to t_26_, while β-myrcene, γ-terpinene, D-limonene, β-phellandrene, and thymol methyl ether remained stable. These terpenes compounds may originate from ingredients such as ginger and garlic [63]. Additionally, sea fennel plant has been previously found to be characterized by an abundance of monoterpenes hydrocarbons in fresh or fermented plants, mainly dominated by limonene, γ-terpinene, and sabinene [28,64]. A recent study conducted by Raffo et al. [65] on the volatile composition of sea fennel from different geographical areas throughout the Italian peninsula identified limonene and thymol methyl ether/γ-terpinene as major compounds. Furthermore, Maoloni et al. [28] reported that the volatile profile of fermented sea fennel undergoes dynamic changes, characterized by an increase and decrease in specific compounds. Similarly, Özcan et al. [66] observed fluctuations in certain terpenic compounds before and after the fermentation of sea fennel plant. These findings suggest that the richness of sea fennel in terpenes likely contributed to the abundance of terpenes in kimchi, alongside contributions from the other ingredients. Sulfur compounds such as dimethylsulfide and trimethylsulfide also showed a significant increase from t_0_ to t_26_. Those results align with Choi et al. [61], who identified dimethylsulfide and trimethylsulfide in both control and started kimchi using different single LAB strains. Jeong and Ko [67] previously reported that dimethylsulfide could be associated with the distinctive sensory properties of fermented kimchi. Those compounds are generally derived from cabbage, red pepper, garlic, and ginger [68]. Kim et al. [69] have previously reported that the activity of LAB during kimchi fermentation, along with the presence of methionine in cabbage, may have contributed to the formation of sulfur-containing volatile aromatic compounds. For instance, dimethyl disulfide provides a sour, rotten, onion-like odor, while dimethyl trisulfide is associated with a cooked cabbage odor [70].

Carboxylic acids, particularly acetic acid, which provides acetic and sour odor notes to kimchi, increased significantly as fermentation progressed. This trend aligns with the results of carboxylic acids quantification reported in Section 2.3 Acetic acid was absent during the early stages of fermentation but became detectable by the late stage (t_26_). This is consistent with findings by Lee et al. [71], who detected acetic acid after 72 h of fermentation, while Shim et al. [23] reported its increase during the mid-term fermentation. Moreover, metabolic compounds such as carboxylic acids, including acetic acid, are crucial for determining the flavor characteristics reflecting the microbial community present in fermented kimchi [72].

At the final stage (t_150_), the volatile profile shifted significantly, with an increase in esters, alcohols, and carboxylic acids. Ethanol and 3-methyl-1-butanol were the most abundant alcohols, highlighting the role of fermentation in enhancing kimchi flavor. These changes align with the findings reported by Hong et al. [59] about increasing ethanol, which accounted for more than 93% of alcoholic compounds during a fermentation conducted at 4 °C in 35 days. However, this increase contrasts with the findings of Lee et al. [71], who observed a decline in alcohol levels during fermentation in a study involving kimchi fermentation at 15 °C with two lactic acid bacteria used as the starter culture. This discordance could be attributed to the ingredients used, the presence of starter, or the fermentation temperature. In general, the formation of alcohol and esters derives from the fatty acids precursors present in the cabbage [73]. For instance, Hong et al. [59] observed no esters in kimchi fermented at 4 °C, while esters were detected in kimchi fermented at 15 °C. These findings suggest that, regardless of the starter culture, environmental factors such as temperature and ingredients play a significant role in generating the final volatile profile of Kimchi.

In addition, the heatmap and PCA analyses confirmed that fermentation time, rather than the use of starter culture, was the dominant factor in the variation of the VOCs profile. The clustering of samples by fermentation time emphasizes the dynamic change in the volatile composition in both kimchi prototypes. The distinct separation of t_0_, t_26_, and t_150_ samples highlights the transformation of aldehydes and terpenes into alcohols and organic acids as fermentation progresses.

Results on the VOCs profile highlight that both the fermentation process and specific ingredients, such as sea fennel, significantly impact the development of kimchi’s distinctive flavor and aroma profile.

In the sensory analysis, similar trends emerged in both the started and control kimchi after 26 days and again after 150 days of fermentation, with no statistically significant differences reported between the two samples at t_26_ and t_150_. However, higher scores were observed for spiciness, chili aroma, and garlic odor on day 26, contributing to the distinctive savory flavor of kimchi, previously described by Lee et al. [18]. These findings align with those of Moon et al. [48], who reported no significant differences in sensory attributes between spontaneously fermented and started kimchi. This similarity may be related to the metabolic activity of lactic acid bacteria, which convert cabbage sugars into organic acids, volatile compounds, and other fermentation by-products that enhance flavor properties [38,74]. Spiciness, generally attributed to capsaicin in red pepper powder, was highly perceived in both samples at the end of fermentation, with a slight decrease after 150 days. Moreover, red pepper is commonly used in kimchi seasonings to enhance flavor and reduce spoilage microorganisms without inhibiting fermenting microbes [75,76]. On the other hand, garlic is considered an essential ingredient in kimchi, providing carbohydrates and nutrients that enhance lactic acid bacteria growth [10].

Crunchiness and hardness were highly perceived in both samples on day 26, but showed a slight decrease after 150 days, reflecting the intrinsic textural qualities of Chinese cabbage [77]. In contrast, the perception of fibrousness increased in both samples over the same period, while the acidic flavor remained consistent throughout the monitoring period. Similarly, Kim et al. [78] observed a progressive decrease in hardness after 35 days of fermentation, suggesting that hardness can be influenced by factors such as the season, manufacturing processes, and leaf position on the cabbage. Furthermore, the aroma of sea fennel was more distinct in started kimchi after 150 days of fermentation, likely due to volatile compounds such as α-pinene and sabinene [79]. Thus, the present study suggests that the interaction between the starter culture and sea fennel influenced the sensory profile.

Regarding overall acceptance, panelists preferred the control kimchi at the end of fermentation, while a higher score was attributed to the started kimchi after 150 days. In this regard, a study on the application of starter cultures to improve the final quality of kimchi carried out by Chang and Chang [26] found that the starter culture significantly enhanced the texture and flavor during fermentation and ripening.

Overall, these results suggest that the application of a defined multi-strain lactic acid bacteria starter culture promotes early lactic acid bacteria dominance in sea fennel kimchi. However, as fermentation progresses, microbial communities in both starter-inoculated and control samples tend to stabilize at similar levels, indicating that starter culture application has minimal influence on the final lactic acid bacteria population structure under the tested conditions.

## 4. Materials and Methods

### 4.1. Starter Formulation

A starter culture, previously optimized for the fermentation of sea fennel sprouts in saline brine [28], was tested in kimchi fermentation trials alongside a naturally fermented control for comparison. The culture consisted of four lactic acid bacteria strains: *Lactiplantibacillus plantarum* (strain PB257), *Leuconostoc pseudomesenteroides* (strain PB288), *Pediococcus pentosaceus* (strain FF78), and *Weissella confusa* (strain PB321). All strains were obtained from the Culture Collection of the Department of Agricultural, Food, and Environmental Sciences (D3A), Università Politecnica delle Marche (Ancona, Italy). Strains were stored at –80 °C in de Man Rogosa and Sharpe (MRS) broth supplemented with glycerol at a 3:2 ratio (broth:glycerol, *v*/*v*). Prior to use, the strains were sub-cultured in MRS broth (VWR) and incubated at 30 °C for 24 h.

### 4.2. Sea Fennel Pre-Treatment

Young leaves and sprouts of sea fennel were supplied by a local farm (Paccasassi del Conero, Castelfidardo, Ancona, Italy), which regularly cultivates organic sea fennel for food applications. Approximately 10 kg of sea fennel sprouts and young leaves were manually harvested. Damaged or woody parts were removed directly in the field, and the edible portions were transported to a local food company (Rinci Srl, Castelfidardo, Ancona, Italy). There, the plant material was thoroughly washed with tap water, air-dried at room temperature, blanched at 95 °C for 30 s, drained for 5 min using an industrial stainless-steel vegetable strainer basket, and then prepared for use in the fermentation trials.

### 4.3. Kimchi Manufacturing

Kimchi was prepared at a local company (Rinci Srl, Castelfidardo, Ancona, Italy), using the ingredients listed in Table 6 and following the process described below. Briefly, approximately 10 kg of Chinese cabbage (*Brassica rapa* subsp. *pekinensis*) was first quartered and then cut into strips approximately 4 cm wide. The outer leaves were removed prior to cutting. After trimming and processing, the total cabbage weight was reduced to 9.3 kg.

The sliced cabbage was washed and soaked for 14 h in a brine solution composed of 40.6 kg of water and 5 kg of NaCl. Following the brining, the cabbage was rinsed twice by immersion in 100 L of tap water, yielding a final weight of 7.5 kg. This was then divided into two equal portions of 3.725 kg each.

A seasoning sauce was prepared by mixing the ingredients listed in Table 6 and stored under refrigeration at +5 °C for approximately 14 h. The sauce was then evenly applied to the cabbage. One portion was mixed with sauce inoculated with the bacterial starter culture, as described in Section 4.1, to achieve a final bacterial load of 7 Log CFU g^−1^ of kimchi. The second portion was mixed with the non-inoculated sauce and served as the control (naturally fermented kimchi).

**Figure 4:**
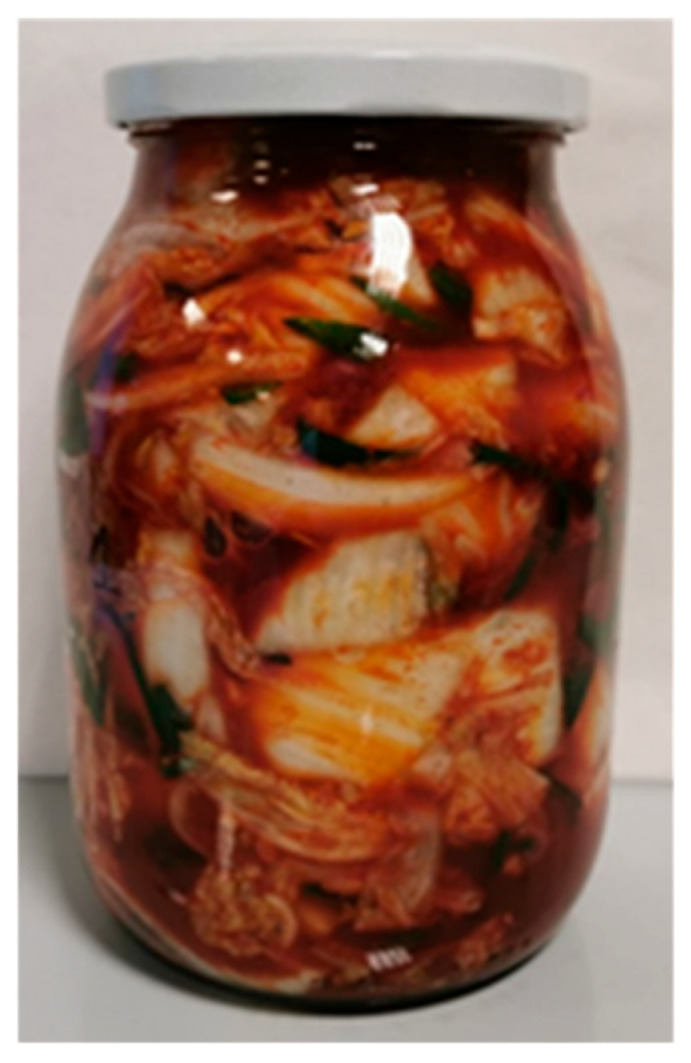
Unconventional kimchi made with the ingredients detailed in Table 1.

Three biological replicates were prepared for both the starter-inoculated and the control kimchi. Each replicate consisted of 1 kg of kimchi placed in a 2 L glass jar. Fermentation was carried out in these jars for 26 days at 4 °C (Figure 4).

### 4.4. Physicochemical Analyses

Aliquots (1 mL) of kimchi sauce were aseptically collected immediately after inoculation (day 0) and at subsequent sampling points during the fermentation process: days 2, 5, 12, 19, and 26. pH measurements were performed using a digital pH meter (Model 300; Hanna Instruments, Padova, Italy). Results were expressed as the mean ± standard deviation of three biological replicates per sample.

For titratable acidity (TA) determination, 10 g aliquots of each biological replicate were weighed and homogenized with 90 mL of deionized water using a Stomacher 400 Circulator (VWR International PBI, Milan, Italy) at 260 rpm for 5 min. The homogenates were then titrated with 0.1 N NaOH until a pH of 8.3 was reached. The results were expressed as percentage of lactic acid equivalents (%) according to Rampanti et al. [80]. Values were reported as the mean ± standard deviation from three biological replicates.

Organic acid determination was performed on deproteinized and decolorized kimchi samples. For deproteinization, a multi-step Carrez clarification procedure was applied: (i) Carrez I solution was prepared by dissolving 3.6 g of potassium hexacyanoferrate (ii) {K_4_[Fe(CN)_6_]·3H_2_O} (Sigma-Aldrich, Milan, Italy). The concentrations of acetic acid and lactic acid were quantified using the Acetic Acid (Acetate Kinase Rapid Manual Format) Assay Kit and the D-/L-Lactic Acid (D-/L-Lactate) (Rapid) Assay Kit (Megazyme, Bray, Ireland). Analyses were conducted on samples collected at days 0, 26, and 150. Results were expressed as means ± standard deviations of three biological replicates.

### 4.5. Microbiological Analyses

Microbial counts were performed on 10 g aliquots of kimchi. Samples were homogenized in 90 mL of sterile peptone water (1 g L^−1^ bacteriological peptone; Oxoid, Basingstoke, UK) using a Stomacher 400 Circulator (International PBI, Milan, Italy) for 2 min at 260 rpm. The homogenates were serially diluted and plated onto selective media for the enumeration of the following microbial groups: (i) Total mesophilic aerobes on Plate Count Agar (PCA), incubated at 30 °C for 48 h; (ii) Presumptive lactobacilli on de Man, Rogosa, and Sharpe (MRS) agar supplemented with cycloheximide (100 mg L^−1^; VWR), incubated at 30 °C for 48 h; (iii) Presumptive mesophilic cocci on M17 agar, incubated at 30 °C for 48 h; (iv) *Enterobacteriaceae* on Violet Red Bile Glucose Agar (VRBGA), incubated at 37 °C for 24 h; (v) *Pseudomonadaceae* on Pseudomonas Agar Base (PAB) supplemented with Cetrimide, Fusidic acid, and Cephaloridine (CFC), incubated at 30 °C for 48 h; (vi) Yeasts and molds on Rose Bengal (RB) agar, incubated at 25 °C for 72 h. Aseptic samples of kimchi were collected from each replicate of the laboratory-scale fermentation prototypes at the end of fermentation and after 150 days using sterilized stainless-steel tweezers. These samples were analyzed for the following: (i) Coagulase-positive *Staphylococcus* spp., using the standard method TEMPO (ISO 6888-2:2021) [81]; (ii) Sulfite-reducing bacteria, according to ISO 15213:2003 [82]; (iii) *Listeria monocytogenes*, using the miniVIDAS system (bioMérieux, Marcy l’Étoile, France) based on the enzyme-linked fluorescent assay (ELFA) method, in accordance with ISO 11290-1 [83,84]. 

### 4.6. Determination of Volatile Compounds via Headspace Solid-Phase MicroExtraction–Gas Chromatography/Mass Spectrometry (HS/SPME-GC/MS)

Ten grams of each homogenized kimchi sample was treated with a calcium chloride dihydrate solution (87 g in 100 mL of deionized water) and homogenized for 1 min using an Ultra-Turrax homogenizer (Ultra -Turrax T25 Digital, IKA-Werke GmbH & Co., Staufen, Germany). The mixture was centrifuged at 2000 rpm for 15 min at 5 °C, and the supernatant was filtered through a Whatman No. 41 filter paper.

Volatile compounds were isolated using Headspace Solid-Phase Microextraction (HS-SPME). Five mL of the filtrate was transferred into a fifteen mL glass vial containing a magnetic stir bar and sealed with a PTFE/silicone septum. The vial was placed in a water bath maintained at 40 °C. A 2 cm, 50/30 µm DVB/CAR/PDMS fiber (Supelco, Sigma-Aldrich, Milan, Italy) was exposed to the headspace for 60 min under stirring (400 rpm) at 40 °C. Following extraction, the fiber was immediately inserted into the GC injector for thermal desorption, and the GC/MS analysis was initiated. The same fiber was used for all analyses to maintain consistency.

GC/MS analysis was performed using an Agilent 6890 GC coupled with a 5973N MS detector equipped with a quadrupole mass filter (Agilent Technologies, Palo Alto, CA, USA). Volatiles were desorbed from the fiber for 3 min in the GC injector at 240 °C in splitless mode. The fiber remained in the injector for an additional 5 min under purge mode (75 mL min^−1^) to remove residual analytes and minimize carry-over. A 0.75 mm SPME liner was installed in the injector.

Separation was achieved using a DB-Wax capillary column (60 m × 0.25 mm i.d., 0.5 µm film thickness) (J&W Agilent Technologies, Santa Clara, CA). The oven temperature program was as follows: 40 °C (held for 10 min), ramped at 4 °C min^−1^ to 235 °C (held for 7 min), with a total runtime of 65.7 min. The inlet and transfer line temperatures were set to 240 °C. Helium was used as the carrier gas at a flow rate of 2.0 mL min^−1^ (linear velocity: 36.3 cm s^−1^). The MS detector operated in electron ionization mode (70 eV), with the ion source and quadrupole temperatures set at 230 °C and 150 °C, respectively. Mass spectra were acquired in full-scan mode (*m/z* 33–300).

Compound identification was performed by comparing mass spectra and linear retention indices with those of authentic standards for all determined VOCs (Merck Life Science S.r.l.; Milan, Italy), except for 1-Methoxy-2-propyl acetate, 5-Cyano-1-pentene, 4-ethyl-5-methylthiazole, Thymol methyl ether, Benzene propanenitrile, and Phenethyl isothiocyanate, for which authentic standards were not available. When standards were not available, tentative identification was based on spectral data from the NIST/EPA/NIH Mass Spectral Library and the published literature. LRIs were calculated using a series of linear alkanes (C_7_–C_30_) under identical chromatographic conditions. For semi-quantitative analysis, duplicate extractions were performed, and VOC levels were estimated based on chromatographic peak areas [85].

### 4.7. Sensory Analysis

Sensory evaluation was conducted according to the method proposed by Maoloni et al. [28], with minor modifications. Assessments were performed at the end of fermentation (day 26) and after ripening (day 150) by a trained panel of eight non-smoking participants (five females, three males; aged 25–48). Panelists were trained to identify and describe sensory attributes specific to kimchi containing sea fennel and to compare starter-inoculated and naturally fermented samples. A preliminary discussion was held to define the most relevant sensory descriptors.

Two kimchi prototypes (starter-inoculated and naturally fermented) were coded with random three-digit numbers and served at room temperature. Panelists evaluated the samples individually in isolated booths. Still bottled water and plain crackers were provided to cleanse the palate between evaluations. Each panelist assessed the samples based on the following sensory attributes: (i) olfactory descriptors: fermented, garlic, pungent, chili, vegetable, sea fennel; (ii) aroma descriptors: fermented, garlic, spicy, chili, vegetable, sea fennel; (iii) flavor descriptors: acidity, bitterness, saltiness, sweetness; (iv) textural descriptors: hardness, fibrousness, crunchiness; (v) overall acceptability.

Aliquots (10 g) of each sample were placed in blind-labeled white plastic cups. Panelists rated the intensity of each descriptor using a 9-point scale (1 = lowest intensity, 9 = highest intensity). Overall preference was assessed using a 9-point hedonic scale, where 1 indicated “extreme dislike” and 9 indicated “extreme like” [86]. Results were reported as mean ± standard deviation for each descriptor across the eight panelists.

### 4.8. Statistical Analysis

One-way analysis of variance (ANOVA) was conducted using JMP software (version 11.0.0; SAS Institute Inc., Cary, NC, USA), followed by Tukey–Kramer’s Honest Significant Difference (HSD) test to determine statistically significant differences among kimchi samples (*p* < 0.05). Principal Component Analysis (PCA) and heatmap visualization were performed on the volatile organic compounds (VOCs) dataset using XLSTAT software (version 2020.1.1; Addinsoft, New York, NY, USA).

## 5. Conclusions

The results collected in this study provide a comprehensive characterization of kimchi fermentation dynamics when incorporating sea fennel as a novel ingredient and comparing spontaneous and starter-mediated fermentation processes. While early fermentation showed notable differences, the two prototypes exhibited high similarity by the ripening stage.

Starter-inoculated kimchi exhibited significantly higher counts of total lactic acid bacteria and mesophilic aerobic bacteria at the beginning of fermentation. However, microbial loads converged in both prototypes by the end of fermentation and throughout ripening. Populations of *Enterobacteriaceae* and *Pseudomonadaceae* significantly declined in both samples, while yeasts were not detected at any stage, regardless of starter culture application.

Volatile profiles at the end of fermentation and during ripening were predominantly characterized by organic acids, alcohols, terpenes, and sulfur-containing compounds. Sensory analysis revealed no significant differences in sensory attributes between the two fermentation methods. Additionally, the inclusion of sea fennel contributed to the volatile and sensory profiles by introducing specific aromatic compounds.

In conclusion, this study demonstrates that both spontaneous and starter-guided fermentation methods were effective in producing high-quality kimchi enriched with sea fennel, showcasing its potential as a functional ingredient in fermented vegetable products. Further research exploring the synergistic effects of sea fennel with other novel ingredients could contribute to optimizing the flavor, nutritional value, and safety of plant-based fermented foods.

## Figures and Tables

**Table 1:** pH of started and naturally fermented (control) kimchi during fermentation.

Sampling Time (Days)	Started Kimchi	Control Kimchi
t_0_	4.90 ± 0.08 ^b,A^	4.87 ± 0.08 ^c,A^
t_2_	5.20 ± 0.07 ^a,A^	5.30 ± 0.17 ^ab,A^
t_5_	5.30 ± 0.07 ^a,B^	5.51 ± 0.13 ^a,A^
t_7_	4.90 ± 0.10 ^b,B^	5.46 ± 0.13 ^ab,A^
t_9_	4.70 ± 0.15 ^bc,B^	5.49 ± 0.07 ^ab,A^
t_12_	4.40 ± 0.13 ^cd,B^	5.16 ± 0.17 ^bc,A^
t_14_	4.20 ± 0.03 ^d,B^	4.97 ± 0.08 ^c,A^
t_16_	4.10 ± 0.19 ^def,B^	4.57 ± 0.08 ^d,A^
t_19_	4.00 ± 0.05 ^ef,B^	4.19 ± 0.08 ^e,A^
t_22_	3.90 ± 0.02 ^f,B^	3.99 ± 0.01 ^e,A^
t_26_	3.90 ± 0.02 ^f,B^	3.97 ± 0.01 ^e,A^

Values are expressed as means ± standard deviations. Superscripts indicate statistical significance according to ANOVA and Tukey’s post hoc test. Superscript lowercase letters indicate statistically significant differences between samples within the same column (*p* < 0.05), while superscript uppercase letters indicate statistically significant differences between samples within the same row (*p* < 0.05).

**Table 2:** Titratable acidity (TA) of started and naturally fermented (control) kimchi during fermentation.

Sampling Time (Days)	Started Kimchi	Control Kimchi
t_0_	0.09 ± 0.01 ^b,A^	0.09 ± 0.01 ^b,A^
t_26_	0.53 ± 0.05 ^a,A^	0.34 ± 0.03 ^a,B^

Values are expressed as means ± standard deviations of % lactic acid equivalents. Superscripts indicate statistical significance according to ANOVA and Tukey’s post hoc test. Superscript lowercase letters indicate statistically significant differences between samples within the same column (*p* < 0.05), while superscript uppercase letters indicate statistically significant differences between samples within the same row (*p* < 0.05).

**Table 3:** Organic acids assessed in started and control kimchi at t_0_ and t_26_ of fermentation_,_ and at t_150_ since the start of fermentation (ripening).

Sampling Time (Days)	Prototype	Lactic Acid			Acetic Acid
		D-Lactic Acid	L-Lactic Acid	Total Lactic Acid	
t_0_	Control kimchi	0.00 ± 0.00 ^c,A^	0.02 ± 0.00 ^b,B^	0.02 ± 0.00 ^b,A^	0.00 ± 0.00 ^b,C^
	Started kimchi	0.01 ± 0.00 ^b,A^	0.02 ± 0.00 ^b,A^	0.03 ± 0.00 ^b,A^	0.00 ± 0.00 ^b,C^
t_26_	Control kimchi	0.19 ± 0.01 ^b,A^	0.38 ± 0.01 ^a,A^	0.56 ± 0.01 ^a,A^	0.02 ± 0.01 ^b,B^
	Started kimchi	0.14 ± 0.01 ^a,B^	0.37 ± 0.03 ^a,A^	0.52 ± 0.04 ^a,A^	0.10 ± 0.02 ^a,A^
t_150_	Control kimchi	0.21 ± 0.01 ^a,A^	0.37 ± 0.02 ^a,A^	0.58 ± 0.03 ^a,A^	0.10 ± 0.02 ^a,A^
	Started kimchi	0.21 ± 0.05 ^a,A^	0.48 ± 0.11 ^a,A^	0.69 ± 0.15 ^a,A^	0.13 ± 0.04 ^a,A^

The results (expressed as g 100 g^−1^ of kimchi) are presented as means ± standard deviations of three biological replicates. Within the same column, for the same compound and sample across different sampling times, means with different lowercase letters indicate significant differences (*p* < 0.05). Similarly, for the same compound and sampling time across different samples, means with different uppercase letters indicate significant differences (*p* < 0.05).

**Table 4:** Viable counts of started and naturally fermented (control) kimchi.

Microbial Group	Sampling Time (Days)	Started Kimchi	Control Kimchi
Mesophilic lactobacilli	t_0_	7.3 ± 0.1 ^b,A^	1.9 ± 0.1 ^d,B^
	t_2_	7.5 ± 0.1 ^b,A^	3.5 ± 0.3 ^c,B^
	t_5_	7.6 ± 0.2 ^b,A^	5.1 ± 0.5 ^b,B^
	t_12_	8.3 ± 0.0 ^a,A^	8.2 ± 0.1 ^a,A^
	t_19_	8.4 ± 0.1 ^a,A^	8.5 ± 0.1 ^a,A^
	t_26_	8.4 ± 0.1 ^a,A^	8.5 ± 0.0 ^a,A^
Mesophilic lactococci	t_0_	7.3 ± 0.1 ^b,A^	4.9 ± 0.1 ^b,B^
	t_2_	7.4 ± 0.0 ^b,A^	5.9 ± 0.3 ^a,B^
	t_5_	7.6 ± 0.2 ^b,A^	5.8 ± 0.3 ^a,B^
	t_12_	8.3 ± 0.0 ^a,A^	6.6 ± 0.3 ^a,B^
	t_19_	8.4 ± 0.1 ^a,A^	6.0 ± 0.5 ^a,B^
	t_26_	8.3 ± 0.1 ^a,A^	6.1 ± 0.4 ^a,B^
Yeasts	t_0_	<1.0 ^a,A^	<1.0 ^a,A^
	t_2_	<1.0 ^a,A^	<1.0 ^a,A^
	t_5_	<1.0 ^a,A^	<1.0 ^a,A^
	t_12_	<1.0 ^a,A^	<1.0 ^a,A^
	t_19_	<1.0 ^a,A^	<1.0 ^a,A^
	t_26_	<1.0 ^a,A^	<1.0 ^a,A^
*Enterobacteriaceae*	t_0_	4.4 ± 0.5 ^a,A^	4.3 ± 0.4 ^a,A^
	t_2_	5.6 ± 0.2 ^a,A^	5.7 ± 1.0 ^a,A^
	t_5_	5.5 ± 0.8 ^a,A^	5.1 ± 0.2 ^a,A^
	t_12_	4.4 ± 0.8 ^a,B^	5.7 ± 0.2 ^a,A^
	t_19_	1.9 ± 0.7 ^b,A^	3.1 ± 0.7 ^b,A^
	t_26_	<1.0 ^c,A^	<1.0 ^c,A^
Mesophilic aerobic bacteria	t_0_	7.2 ± 0.0 ^c,A^	5.4 ± 0.1 ^b,B^
	t_2_	7.5 ± 0.1 ^bc,A^	6.2 ± 0.7 ^b, B^
	t_5_	7.6 ± 0.3 ^b,A^	5.8 ± 0.3 ^b,B^
	t_12_	8.3 ± 0.0 ^a,A^	8.3 ± 0.1 ^a,A^
	t_19_	8.4 ± 0.1 ^a,A^	8.6 ± 0.1 ^a,A^
	t_26_	8.4 ± 0.0 ^a,A^	8.4 ± 0.2 ^a,A^
*Pseudomonadaceae*	t_0_	5.2 ± 0.3 ^a,A^	5.2 ± 0.1 ^a,A^
	t_2_	5.0 ± 0.3 ^a,A^	5.7 ± 0.6 ^a,A^
	t_5_	4.9 ± 0.3 ^a,A^	5.1 ± 0.6 ^a,A^
	t_12_	3.4 ± 0.3 ^b,B^	5.2 ± 0.2 ^a,A^
	t_19_	2.5 ± 0.6 ^bc,A^	2.8 ± 0.3 ^b,A^
	t_26_	2.1 ± 0.4 ^c,A^	2.7 ± 0.6 ^b,A^

Values are expressed as Log CFU g^−1^ ± standard deviation of three biological replicates. Within each row, for each microbial group at the same sampling time, overall means with uppercase superscript letters are significantly different (*p* < 0.05). For each microbial group and sample, overall means with different lowercase superscript letters in the same column are significantly different (*p* < 0.05).

**Table 5:** Volatile compounds (area of chromatographic peak, mean ± SD; n = 3) detected in control and started kimchi made with sea fennel during fermentation and ripening.

N°	Retention Index	Volatile Compound	Peak Area (×10^5^)					
			Control Kimchi			Started Kimchi		
			t_0_	t_26_	t_150_	t_0_	t_26_	t_150_
1	705	Acetaldehyde	2.57 ± 0.33 ^a^	0.00 ± 0.00 ^b^	1.18 ± 2.05 ^b^	3.48 ± 0.21 ^a^	0.00 ± 0.00 ^b^	0.00 ± 0.00 ^b^
2	822	Ethyl Acetate	11.39 ± 2.23 ^b^	23.27 ± 2.61 ^b^	38.58 ± 18.61 ^ab^	13.12 ± 1.83 ^b^	37.69 ± 6.65 ^ab^	274.85 ± 388.89 ^a^
3	950	Ethanol	30.19 ± 3.50 ^c^	78.53 ± 1.95 ^bc^	175.14 ± 89.02 ^a^	34.53 ± 4.38 ^c^	64.34 ± 3.55 ^c^	124.80 ± 12.66 ^ab^
4	987	Pentanal	0.00 ± 0.00 ^b^	0.00 ± 0.00 ^b^	0.00 ± 0.00 ^b^	4.80 ± 1.16 ^a^	3.80 ± 1.08 ^a^	0.00 ± 0.00 ^b^
5	1029	α-Pinene	3.27 ± 0.15 ^ab^	3.48 ± 0.77 ^a^	1.28 ± 1.34 ^bc^	4.19 ± 2.29 ^a^	4.13 ± 0.81 ^a^	1.09 ± 1.01 ^c^
6	1076	Camphene	3.62 ± 1.66 ^ab^	4.23 ± 1.18 ^ab^	3.57 ± 1.11 ^ab^	3.62 ± 1.07 ^ab^	5.54 ± 1.27 ^a^	3.32 ± 1.73 ^b^
7	1086	Dimethyl disulfide	21.22 ± 9.37 ^c^	59.55 ± 19.50 ^a^	43.41 ± 7.56 ^ab^	27.45 ± 10.87 ^bc^	57.47 ± 17.08 ^a^	25.71 ± 6.78 ^bc^
8	1128	Sabinene	30.91 ± 24.26 ^a^	0.52 ± 0.90 ^b^	0.00 ± 0.00 ^b^	20.16 ± 3.41 ^a^	0.00 ± 0.00 ^b^	0.00 ± 0.00 ^b^
9	1135	1-Butanol-3-methyl acetate	0.00 ± 0.00 ^b^	0.00 ± 0.00 ^b^	4.03 ± 3.54 ^a^	0.00 ± 0.00 ^b^	0.00 ± 0.00 ^b^	18.49 ± 21.32 ^a^
10	1172	β-Myrcene	2.39 ± 0.99 ^a^	2.64 ± 2.3 ^a^	1.77 ± 1.54 ^a^	3.03 ± 1.29 ^a^	2.61 ± 2.27 ^a^	1.31 ± 1.13 ^a^
11	1177	1-Penten-3-ol	4.07 ± 1.33 ^c^	15.21 ± 1.58 ^b^	13.87 ± 7.26 ^b^	5.32 ± 1.40 ^c^	27.83 ± 2.74 ^a^	15.07 ± 2.60 ^b^
12	1209	D-Limonene	54.76 ± 31.07 ^a^	92.47 ± 26.27 ^a^	73.37 ± 25.59 ^a^	98.69 ± 64.87 ^a^	109.32 ± 18.69 ^a^	83.08 ± 21.47 ^a^
13	1220	β-Phellandrene	15.34 ± 2.28 ^ab^	22.30 ± 5.85 ^a^	12.84 ± 1.79 ^b^	21.78 ± 7.35 ^a^	23.92 ± 3.86 ^a^	12.56 ± 2.02 ^b^
14	1226	3-Methyl-1-butanol	0.19 ± 0.33 ^b^	1.24 ± 2.14 ^b^	275.84 ± 239.17 ^a^	0.21 ± 0.36 ^b^	2.57 ± 2.23 ^b^	200.60 ± 101.87 ^a^
15	1226	1,8-Cineol	11.23 ± 0.67 ^bc^	18.19 ± 4.91 ^ab^	7.18 ± 12.43 ^bc^	16.14 ± 4.70 ^ab^	23.91 ± 1.98 ^a^	3.64 ± 6.31 ^c^
16	1238	1-Methoxy-2-propyl acetate	74.98 ± 38.38 ^a^	99.21± 4.15 ^a^	79.62 ± 11.03 ^a^	81.61 ± 33.90 ^a^	80.96 ± 18.71 ^a^	73.02 ± 9.87 ^a^
17	1260	γ-Terpinene	86.98 ± 38.29 ^a^	137.06 ± 35.79 ^a^	105.65 ± 26.25 ^a^	125.27 ± 60.17 ^a^	140.77 ± 17.00 ^a^	107.74 ± 29.15 ^a^
18	1267	1-Pentanol	3.67 ± 3.18 ^c^	5.60 ± 4.85 ^c^	12.90 ± 2.08 ^ab^	4.23 ± 1.39 ^c^	8.37 ± 3.37 ^bc^	17.54 ± 1.68 ^a^
19	1286	p-Cymene	56.97 ± 12.26 ^c^	109.89 ± 20.52 ^a^	79.04 ± 17.95 ^abc^	79.08 ± 33.77 ^abc^	105.70 ± 8.44 ^ab^	73.83 ± 14.52 ^bc^
20	1367	5-Cyano-1-pentene	39.13 ± 10.03 ^bc^	38.29 ± 5.87 ^c^	33.48 ± 3.27 ^c^	51.90 ± 9.10 ^a^	58.34 ± 5.05 ^a^	49.91 ± 3.08 ^ab^
21	1402	(Z)-3-Hexen-1-ol	29.19 ± 7.54 ^b^	41.61 ± 7.42 ^a^	40.86 ± 5.61 ^a^	39.41 ± 1.57 ^a^	45.92 ± 4.73 ^a^	46.20 ± 8.25 ^a^
22	1408	Dimethyl trisulfide	8.19 ± 4.94 ^bc^	21.82 ± 11.67 ^a^	8.98 ± 9.62 ^bc^	13.73 ± 4.64 ^abc^	15.36 ± 3.91 ^ab^	2.68 ± 2.35 ^c^
23	1467	Acetic acid	1.59 ± 1.40 ^b^	74.40 ± 2.76 ^ab^	57.71 ± 12.00 ^ab^	2.55 ± 0.40 ^b^	133.76 ± 116.89 ^a^	120.34 ± 106.02 ^a^
24	1556	Camphor	49.19 ± 3.98 ^a^	47.67 ± 2.77 ^a^	46.47 ± 3.64 ^a^	35.85 ± 24.45 ^a^	34.67 ± 12.92 ^a^	41.68 ± 3.32 ^a^
25	1564	4-ethyl-5- methlylthiazole	40.39 ± 10.76 ^cd^	72.08 ± 15.56 ^ab^	52.04 ± 2.68 ^bc^	65.80 ± 5.27 ^abc^	79.47 ± 13.91 ^a^	21.66 ± 31.09 ^d^
26	1614	Thymol methyl ether	63.06 ± 17.06 ^a^	91.32 ± 17.40 ^a^	66.80 ± 5.48 ^a^	108.83 ± 64.53 ^a^	99.14 ± 6.01 ^a^	71.04 ± 15.74 ^a^
27	1630	Terpinene-4-ol	54.12 ± 24.75 ^d^	239.30 ± 41.02 ^c^	451.32 ± 20.36 ^a^	49.23 ± 5.76 ^d^	249.58 ± 28.29 ^c^	333.30 ± 15.01 ^b^
28	1705	Neral	5.00 ± 1.79 ^ab^	4.30 ± 4.02 ^ab^	1.63 ± 2.82 ^b^	8.02 ± 1.92 ^a^	5.27 ± 0.71 ^ab^	1.50 ± 2.61 ^b^
29	1719	α-Terpineol	10.77 ± 2.75 ^c^	28.95 ± 5.14 ^ab^	26.32 ± 0.74 ^b^	11.35 ± 1.65 ^c^	32.07 ± 0.96 ^a^	30.62 ± 3.15 ^ab^
30	1730	Borneol	9.13 ± 1.13 ^b^	11.18 ± 2.68 ^ab^	10.43 ± 2.71 ^ab^	11.30 ± 2.43 ^ab^	13.58 ± 0.85 ^a^	11.76 ± 3.81 ^ab^
31	1757	Geranial	23.81± 2.44 ^ab^	24.70 ± 14.88 ^ab^	21.71 ± 7.18 ^ab^	27.24 ± 6.38 ^a^	24.14 ± 1.82 ^ab^	11.94 ± 10.44 ^b^
32	1864	Hexanoic acid	0.40 ± 0.70 ^c^	8.59 ± 0.80 ^b^	17.03 ± 1.74 ^a^	0.00 ± 0.00 ^c^	7.69 ± 1.70 ^b^	17.76 ± 7.04 ^a^
33	1946	Phenylethyl alcohol	0.38 ± 0.70 ^c^	3.06 ± 2.65 ^bc^	37.23 ± 28.23 ^a^	0.65 ± 0.62 ^c^	4.86 ± 0.49 ^bc^	18.98 ± 0.85 ^ab^
34	2080	Octanoic acid	0.96 ± 1.66 ^cd^	10.47 ± 9.06 ^bc^	39.17 ± 5.25 ^a^	0.00 ± 0.00 ^d^	16.79 ± 3.03 ^b^	41.14 ± 9.0 3^a^
35	2083	Benzene propanenitrile	21.64 ± 7.55 ^bc^	25.89 ± 5.36 ^ab^	14.63 ± 6.85 ^c^	27.17 ± 2.41 ^ab^	32.86 ± 2.26 ^a^	25.34 ± 2.20 ^ab^
36	2264	Carvacrol	5.47 ± 1.85 ^b^	8.94 ± 1.73 ^a^	7.54 ± 1.06 ^ab^	7.56 ± 0.90 ^ab^	8.71 ± 1.01 ^a^	7.49 ± 0.91 ^ab^
37	2276	Phenethyl isothiocyanate	14.33 ± 3.26 ^c^	23.74 ± 5.65 ^a^	17.04 ± 0.77 ^abc^	15.79 ± 1.30 ^bc^	23.04 ± 3.96 ^ab^	13.57 ± 7.24 ^c^
38	2385	Dillapiole	63.39 ± 19.92 ^bc^	116.94 ± 28.05 ^a^	99.47± 6.48 ^ab^	81.71 ± 20.20 ^abc^	71.68 ± 18.51 ^bc^	57.25 ± 19.23 ^c^

Means with different superscripts within the same row are significantly different (*p* < 0.05). Samples are displayed according to time—t_0_: day of production; t_26_: end of fermentation; t_150_: ripening stage.

**Table 6:** Ingredients used in kimchi preparation.

Ingredient	Weight (g)	% (*w*/*w*)
Chinese cabbage	3725.34	70.53
Onion	230.62	4.37
Garlic	26.61	0.50
Ginger	26.61	0.50
Sea fennel	372.53	7.05
Red pepper	8.87	0.17
Paprika	97.57	1.85
Sugar	44.35	0.84
Salt	39.91	0.76
Water	709.59	13.43

## Data Availability

The original contributions presented in this study are included in the article. Further inquiries can be directed to the corresponding author(s).
